# Screening of Potential *Vibrio cholerae* Bacteriophages for Cholera Therapy: A Comparative Genomic Approach

**DOI:** 10.3389/fmicb.2022.803933

**Published:** 2022-03-29

**Authors:** Ranjan Kumar Barman, Alok Kumar Chakrabarti, Shanta Dutta

**Affiliations:** ^1^Division of Virology, ICMR-National Institute of Cholera and Enteric Diseases, Kolkata, India; ^2^Division of Bacteriology, ICMR-National Institute of Cholera and Enteric Diseases, Kolkata, India

**Keywords:** bacteriophage, phage therapy, AMR, comparative genomics, phylogenetic tree, *Vibrio cholerae*

## Abstract

Cholera continues to be a major burden for developing nations, especially where sanitation, quality of water supply, and hospitalization have remained an issue. Recently, growing antimicrobial-resistant strains of *Vibrio cholerae* underscores alternative therapeutic strategies for cholera. Bacteriophage therapy is considered one of the best alternatives for antibiotic treatment. For the identification of potential therapeutic phages for cholera, we have introduced a comprehensive comparative analysis of whole-genome sequences of 86 *Vibrio cholerae* phages. We have witnessed extensive variation in genome size (ranging from 33 to 148 kbp), GC (G + C) content (varies from 34.5 to 50.8%), and the number of proteins (ranging from 15 to 232). We have identified nine clusters and three singletons using BLASTn, confirmed by nucleotide dot plot and sequence identity. A high degree of sequence and functional similarities in both the genomic and proteomic levels have been observed within the clusters. Evolutionary analysis confirms that phages are conserved within the clusters but diverse between the clusters. For each therapeutic phage, the top 2 closest phages have been identified using a system biology approach and proposed as potential therapeutic phages for cholera. This method can be applied for the classification of the newly isolated *Vibrio cholerae* phage. Furthermore, this systematic approach might be useful as a model for screening potential therapeutic phages for other bacterial diseases.

## Introduction

Cholera is an acute diarrheal disease caused by *Vibrio cholerae*, a gram-negative water-borne bacterium, and it remains to be a major global health problem. It is endemic to several parts of Asia, Africa, and South and Central America (Zuckerman et al., 2007). Oral and intravenous rehydration therapy is practiced for treating mild and severe cholera patients. A number of antibiotics are also recommended for mild and severe cases. In the past, several antibiotics including doxycycline, tetracycline, ciprofloxacin, and azithromycin have been utilized to treat cholera patients (Sack et al., 1978; Islam, 1987; Gotuzzo et al., 1995; Hossain et al., 2002; Saha et al., 2006). However, recently, treatment failures are often observed due to the emergence of antimicrobial resistance (AMR) in *Vibrio cholerae* (*V. cholerae*) (Verma et al., 2019; Das et al., 2020). This underscores the alternative approach for cholera therapy. Therefore, recently, scientific communities are more focused on phage therapy as an alternative to antibiotics, especially where AMR is a problem (Czaplewski et al., 2016; Rohde et al., 2018; D’Accolti et al., 2021; Froissart and Brives, 2021). Phages are specific in their targets whereas antibiotics target a broad spectrum of both pathogenic and non-pathogenic microorganisms. In phage therapy, no severe side effects have been observed and fewer doses are required (Loc-Carrillo and Abedon, 2011; Principi et al., 2019).

Several experimental approaches have been offered for the isolation and characterization of bacteriophages (Thanki et al., 2019; Bujak et al., 2020; Kwon et al., 2020; McCutcheon et al., 2020; Sofy et al., 2021). Similarly, many *Vibrio cholerae* phages have also been isolated and characterized (Nesper et al., 1999; Faruque et al., 2005a,b; Nelson et al., 2009; Seed et al., 2011; Comeau et al., 2012; Das et al., 2012; Li et al., 2013; Solis-Sanchez et al., 2016; Naser et al., 2017). So far only a few *Vibrio cholerae* phages were studied in animal models which have been proven effective to protect against cholera challenge and may be useful to treat cholera (Yen et al., 2017; Bhandare et al., 2019). Bhandare et al. (2019) presented a bacteriophage Phi_1 to control cholera in an infant rabbit model. They showed that the oral administration of Phi_1 phage can reduce bacterial load significantly (Bhandare et al., 2019). Yen et al. (2017) tested a cocktail of three phages (ICP1, ICP2, and ICP3) to prevent cholera in infant mouse and rabbit models. They revealed that oral administration of a cocktail of three phages significantly reduced both colonization and cholera-like diarrhea (Yen et al., 2017). On the other hand, the sequencing cost of the whole genome is significantly reduced due to the advancement in sequencing technology. As a result, several whole-genome sequences of bacteriophages and their detailed characterization are available in the public domain. Comparative genomic methods might be helpful for the screening of potential bacteriophages for therapy. Recently, several comparative genomic studies have been offered to understand the phages and their clinical implication on host bacteria (Fouts et al., 2013; Turner et al., 2017; Ha and Denver, 2018; Gao et al., 2020). Fouts et al. (2013) compared two *Vibrio cholerae* O139 Bengal-specific phages to understand the genetic and structural differences among them. They observed that 59 out of 79 predicted proteins are identical, and there were few SNP (single-nucleotide polymorphisms) and small INDEL (insertions/deletions) among the two phages (Fouts et al., 2013). Gao et al. (2020) analyzed the whole-genome sequence of 142 prophages of *Salmonella enterica* and classified 17 discrete clusters for 90 phages and 52 singletons. They have noticed high diversity among the phages that and might help the practical utilization of phages as antibacterial agents (Gao et al., 2020). Turner et al. (2017) also examined 37 *Acinetobacter* phages and obtained seven distinct clusters and two singletons. They claim that this study will aid in the classification of novel isolated *Acinetobacter* phages (Turner et al., 2017). Similarly, Ha and Denver (2018) also analyzed 130 complete genome sequences of *Pseudomonas* phages and recognized 12 discrete clusters and 30 singletons. They also reported extensive gene diversity among the phages (Ha and Denver, 2018). Angermeyer et al. (2018) compared complete genome sequences of 19 distinct isolates of *Vibrio cholerae* phage ICP1 to comprehend how ICP1 phage is changing over the years 2001–2012. They found that ICP1 isolates are highly conserved and retain a large core genome. However, over the years, ICP1 also acquired some unknown genes, as well as the CRISPR-Cas system. No comprehensive comparative genomic method is available for critical analysis of *Vibrio cholerae* phages. Therefore, comprehensive comparative genomics analysis of *Vibrio cholerae* phages might help us understand the correlation among them, classification of newly isolated phages, and screening of the potential therapeutic phages.

In the current study, we have introduced a comprehensive comparative genomic approach to get clear genomic, functional characteristics of *Vibrio cholerae* phages and identification of potential phages for cholera therapy. For this purpose, we have examined all the available complete genome sequences of *Vibrio cholerae* phages in the public domain. We have extensively used state-of-the-art comparative genomic analysis methods.

## Materials and Methods

### Genome Sequence of *Vibrio cholerae* Phages

All the 86 complete genome sequences of *Vibrio cholerae* phages were manually curated and downloaded from the Reference Sequence (RefSeq) and the International Nucleotide Sequence Database Collaboration (INSDC) databases of the National Center for Biotechnology Information (NCBI). The available complete genome sequences of phages and stated *Vibrio cholerae* in the host field were manually confirmed and considered for this study (Supplementary Table 1).

### Genome Annotation

To assure annotation evenness, we have randomly selected 10 out of 86 *Vibrio cholerae* phages and re-annotated them. All the possible genes of phage are predicted by GeneMarkS (Besemer and Borodovsky, 2005). The functional annotations of the predicted genes were carried out by NCBI BLASTp program with non-redundant protein sequences (nr) database (Boratyn et al., 2013). Putative transfer ribonucleic acid (tRNA) was predicted by tRNAscan-SE (Lowe and Chan, 2016). For these 10 phages, we have noticed similar structural and functional annotation as reported in NCBI; hence, we have not executed the above process for the remaining phages.

### Clustering of Genome

To determine the complete genome-wise similarity among the phages, we have utilized the BLASTn program of NCBI (Boratyn et al., 2013). Each phage was BLAST against the remaining 85 phages. For assigning the phages in the same genome cluster, we have considered BLASTn query coverage and identity > 75% with an E-value threshold of 0. However, for the majority of clusters we have noticed BLASTn query coverage and identity > 90% with an E-value threshold of 0.

All the complete genome sequences of phages were concatenated into a single nucleotide sequence and considered as an input in nucleotide dot-plots. Genome Pair Rapid Dotter (Gepard) version 1.30 has been employed for comparison of whole-genomes and visualization (Krumsiek et al., 2007). The MUMmer program of JSpecies has been applied for computing average nucleotide identity (ANI) among the phages (Richter et al., 2016; Marcais et al., 2018). Orthologous groups of proteins among the phages were identified by OrthoFinder (Emms and Kelly, 2019). Comparative whole-genome maps of phages are visualized by utilizing CGView (Grant and Stothard, 2008). We have considered the first phage genome of a cluster as a reference genome and BLAST with the other phage genomes (mainly 3–5) in CGView.

### Evolutionary Analysis

Based on the presence of DNA polymerase proteins among the majority of the *Vibrio cholerae* phages, we have considered this protein for evolutionary analysis. The DNA polymerase protein sequence of phages were extracted and stored into a single FASTA file. The FASTA file contains a single DNA polymerase protein of each phage. Furthermore, we have incorporated the DNA polymerase protein of *Klebsiella pneumoniae* and *Salmonella typhi* for outgroup identification. For the evolutionary study, we have utilized the Molecular Evolutionary Genetics Analysis (MEGA) version 10 (X) software (Kumar et al., 2018). Multiple Sequence Comparison by Log-Expectation (MUSCLE) and Unweighted Pair Group Method with Arithmetic Mean (UPGMA) tools of MEGA have been used for protein sequence alignment and phylogenetic tree construction, respectively (Edgar, 2004). The bootstrap value of 2,000 and a 75% cut-off value of a condensed tree was considered for the final construction of the phylogenetic tree.

### Screening of Potential Phage for Therapy

In the recent past, *Vibrio cholerae* phages Phi_1 and ICP cocktail (ICP1, ICP2, and ICP3) have been experimentally used to understand the therapeutic application of cholera phages in the animal model (Yen et al., 2017; Bhandare et al., 2019). For further analysis, we have examined only the cluster that should have at least one therapeutic phage. The whole-genome of phages in a cluster was analyzed by Clustal Omega and MAFET (Katoh et al., 2002; Madeira et al., 2019). We have considered guided tree and identity matrix value to identify the top two similar phages of therapeutic phage. Furthermore, protein sequence level similarity and the orthologous group of proteins among the screening phages were identified by CD-HIT Suite and OrthoFinder, respectively (Huang et al., 2010). Finally, whole-genome data of therapeutic phages and their top two closest phages were analyzed and visualized by Mauve (Darling et al., 2004).

## Results

### Genomic Characteristic

We have investigated a total of 86 complete genomes of *Vibrio cholerae* phages. As presented in Table 1, the genome size of phages varied from 33 to 148 kbp. However, in the majority of phages, genome size was found to be nearly 40 kbp and the GC (G + C) content ranges from 34.5 to 50.8%. Although the number of proteins encoded by these phage genomes varies from 15 to 232, we found that the majority of phage-encoded proteins range from 40 to 50. Five out of 86 phages were found to encode tRNA with a range of 1–17. Vibrio phages JSF10 (NC_042074), phi 3 (NC_028895), vB_VchM_Kuja (NC_048827), JA-1 (NC_021540), and VCO139 (NC_049350) have encoded tRNAs of 17, 8, 3, 1, and 1, respectively.

**TABLE 1:** The genomic characteristic of 86 complete genomes of *Vibrio Cholerae* phages.

Genomic features	Number or range
Complete genome size (bp)	33,106–148,180
GC (%)	34.5–50.8
Proteins	15–232
tRNAs	1–17

### Genome Clusters

**FIGURE 1:**
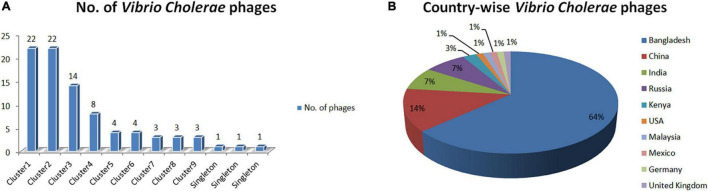
**(A)** Distribution of *Vibrio Cholerae* phages in clusters. **(B)** Isolation country of *Vibrio Cholerae* phages in the world.

The BLASTn sequence similarity among the phages is computed to recognize phage genome clusters. We have identified nine clusters and three singletons of phages using BLASTn sequence coverage and identity > 75% with an E-value threshold of 0.0 (Supplementary Table 2). As displayed in Figure 1A, Clusters 1 and 2 have the highest number of phages (22 for each) and the smallest number of phages (three for each) are found in Clusters 7, 8, and 9. The three singletons are also shown here. Our analysis revealed that the maximum number of phage genome sequences was reported from Bangladesh (64%) followed by China (14%), India (7%), and Russia (7%), respectively (shown in Figure 1B).

**FIGURE 2:**
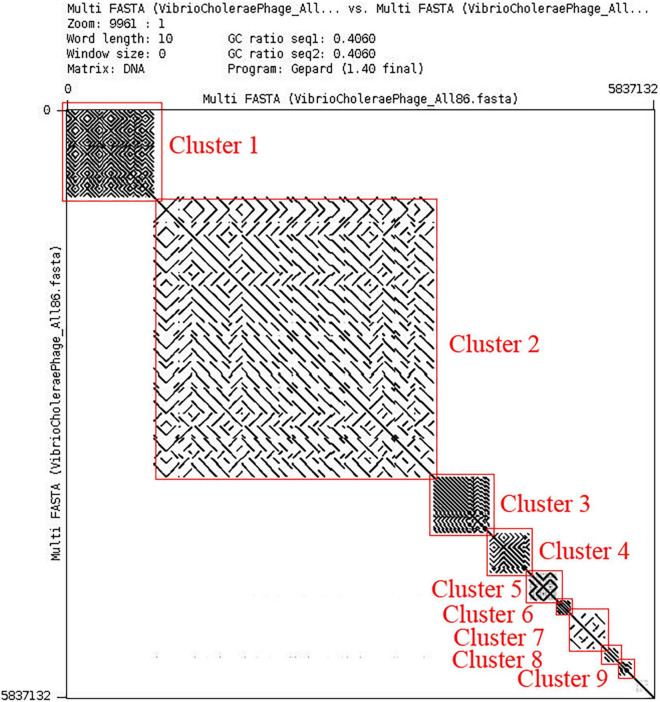
Dot plot of all the 86 *Vibrio Cholerae* phages. Dot plot is visualized by Gepard version 1.30 (Krumsiek et al., 2007).

All possible pairwise sequence similarity among the phages were computed by whole-genome dot plot and ANI. All the 86 whole-genome sequences of phages were concatenated into a single nucleotide file and used for both axes. The exact match of nucleotide was plotted as a black dot. Therefore, parallel black lines to the main diagonal shows continuous and strong sequence similarity whereas gray lines show weak similarity. We have observed the presence of nine clusters and three singletons (shown in Figure 2) by analyzing this dot matrix. Furthermore, we have considered one representing phage for each cluster and three singletons (9 + 3 = 12) and treated it as a ClusterRep (Representing Cluster). All the downstream analyses were also carried out for this cluster. The dot plot analysis for ClusterRep has displayed no similarity among the 12 phages (Supplementary Figure 1).

The ANI among the phages of a respective cluster was computed (Table 2 and Supplementary Tables 3–10) and in most of the cases, we have observed the ANI of phage pairs is > 95%. The ANI of cluster1 ranges from 93.97 to 100%. Similarly, a minimum ANI score of 98.72% and a maximum score of 100% have been observed for Cluster2. A similar ANI pattern was also observed in other clusters. As presented in Table 2, the majority of phage pairs have ANI > 97% in Cluster 4. No similarity was noticed for ClusterRep (Supplementary Table 11).

**TABLE 2:** Average nucleotide identity (ANI) among the phages of Cluster 4.

	ICP2-2013-A-Haiti	Saratov15	Saratov12	ICP2	ICP2-2011-A	ICP2-2006-A	JSF27	JSF23
ICP2-2013-A-Haiti	*	85.79	85.74	86	85.82	85.99	85.99	85.84
Saratov15	85.92	*	99.99	97.61	97.68	97.62	97.66	97.62
Saratov12	85.87	99.99	*	97.57	97.67	97.59	97.63	97.57
ICP2	86.01	97.61	97.57	*	99.05	98.97	99.21	98.96
VICP2-2011-A	85.82	97.68	97.67	99.05	*	99.72	99.81	99.86
ICP2-2006-A	85.99	97.62	97.59	98.97	99.72	*	99.72	99.82
JSF27	85.99	97.66	97.63	99.21	99.81	99.72	*	99.73
JSF23	85.84	97.62	97.57	98.96	99.86	99.82	99.73	*

*specified ANI for same phage.

For a respective cluster, we have detected a substantial number of orthologous groups of proteins. For Clusters 1, 2, 3, 4, 5, 6, 7, 8, and 9, the number of orthologous groups was found to be 54, 269, 38, 35, 101, 44, 152, 70, and 67, respectively (Supplementary Tables 12–20). Interestingly, we have not found any orthologous group of proteins for ClusterRep.

**FIGURE 3:**
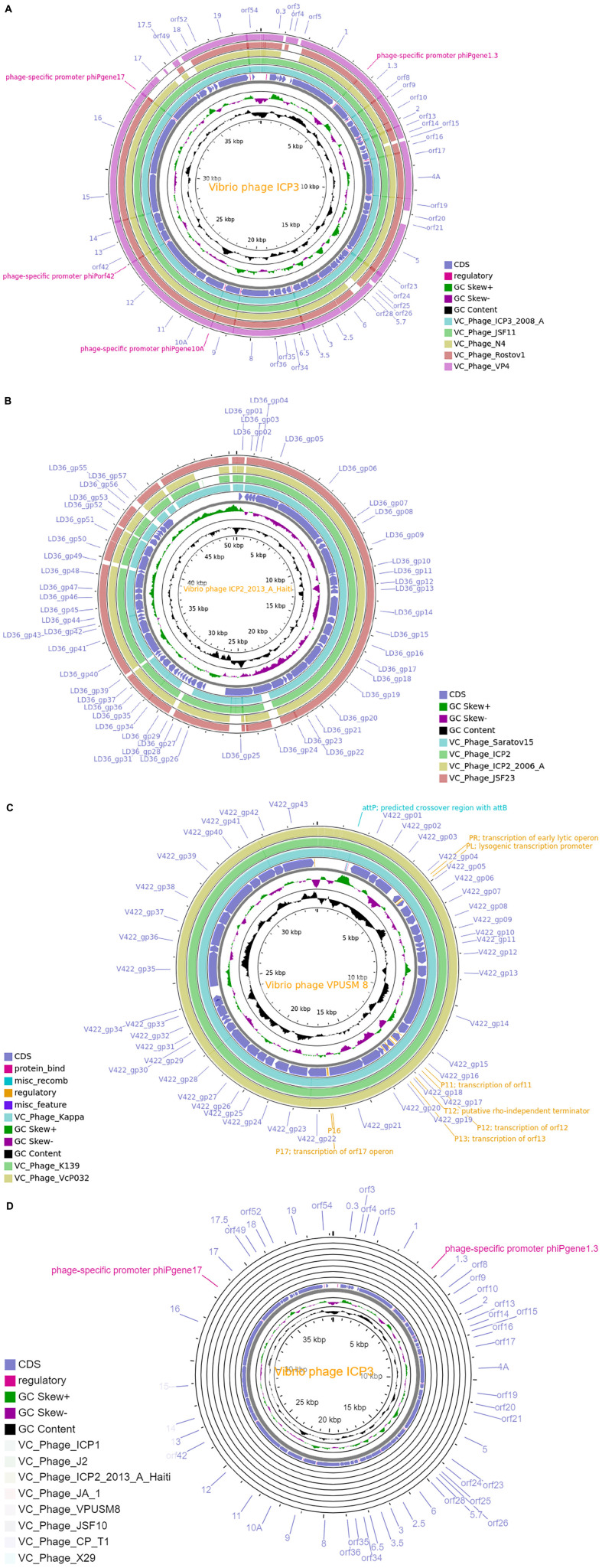
**(A)** Comparative whole-genome map of ICP 3 phage and Cluster 1 phages. **(B)** Comparative whole-genome map of ICP2-2013-A-Haiti phage and Cluster 4 phages. **(C)** Comparative whole-genome map of VPUSM 8 phage and Cluster 6 phages. **(D)** Comparative whole-genome map of ICP 3 phage and ClusterRep phages. All CDS, GC content, and skew of reference genome are also shown in the above figures.

We have employed the CGView tool for the comparative whole-genome map of each cluster along with ClusterRep. As presented in Figures 3A–D and Supplementary Figures 2–7, we have used the first phage of a cluster as a reference genome and BLAST with the other phage within the cluster. The similarities among the phages were plotted by a solid color circle and dissimilarities among the phages were plotted by a white color circle. For Clusters 1–9, high similarities have been observed within the cluster whereas, no similarities were observed in ClusterRep.

### Phylogenetic Analysis

**FIGURE 4:**
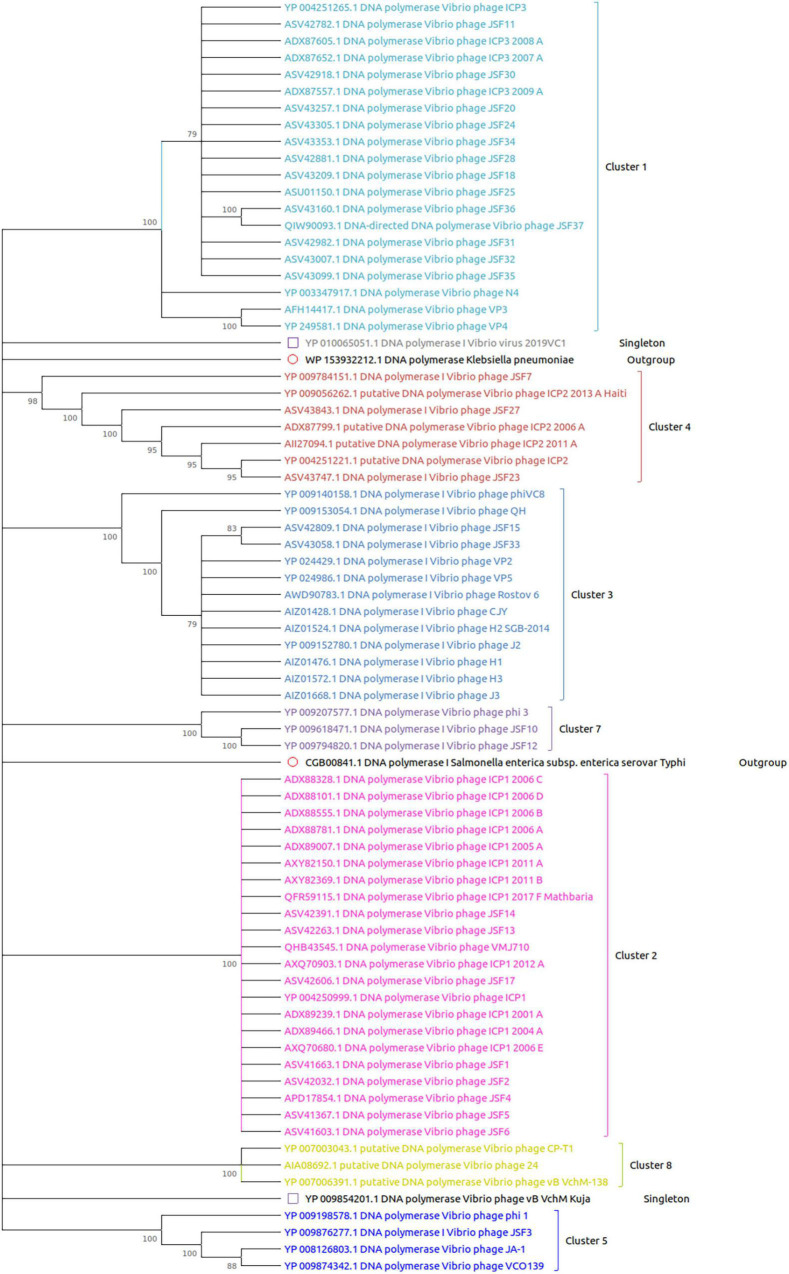
Evolutionary analysis of DNA polymerase proteins. The MEGA X software with MUSCLE, the UPGMA algorithm, the bootstrap value of 2,000, and a 75% cut-off value of a condensed tree have been used for the analysis. The outgroup DNA polymerase proteins are also included for significant analysis.

**FIGURE 5:**
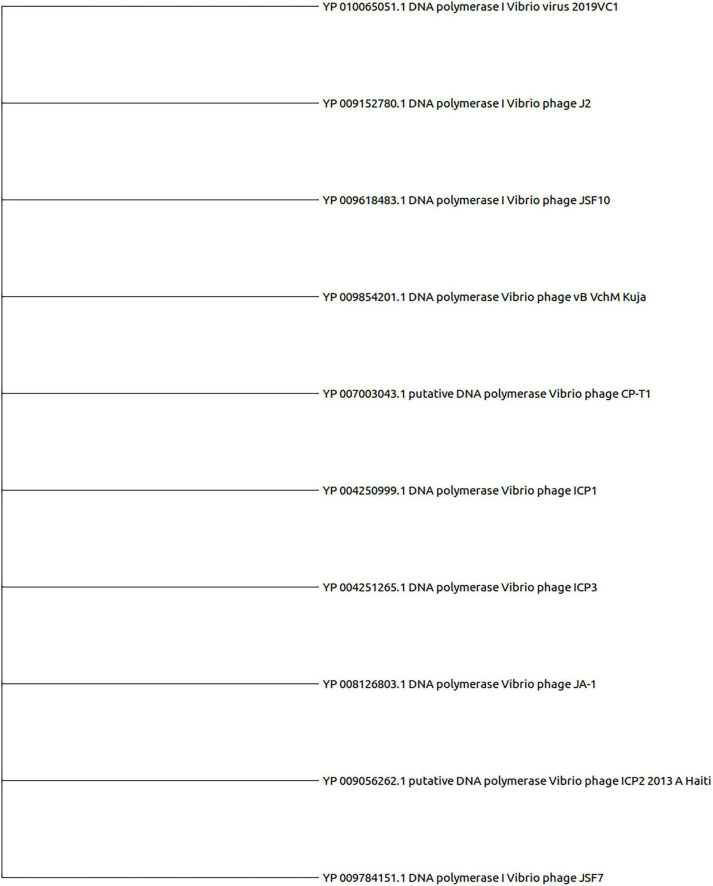
Evolutionary analysis of DNA polymerase proteins of ClusterRep. The MEGA X software with MUSCLE, the UPGMA algorithm, the bootstrap value of 2,000, and a 75% cut-off value of a condensed tree have been used for the analysis.

The DNA polymerase proteins of each phage have been incorporated for the construction of the phylogenetic tree. We have noticed the presence of DNA polymerase protein in the genome of 74 out of 86 phages. As shown in Figure 4, all the clusters and outgroups are distinguishable using evolutionary analysis of DNA polymerase proteins. Figure 4 demonstrates that the phages are highly evolutionary conserved within the cluster. However, for ClusterRep, no evolutionary conservation was found (shown in Figure 5).

### Screening of Potential Phage for Cholera Therapy

To screen potential phages which may be used for therapeutic application, we have investigated those clusters that have at least one therapeutic phage. As a result, we have considered Clusters 1, 2, 4, and 5 for the final analysis. For the ICP3 (NC_015159.1) therapeutic phage in Cluster 1, we have detected ICP3_2009_A (HQ641342.1) and ICP3_2007_A (HQ641344.1) as the top two closest phages (Supplementary Table 21 and Supplementary Figure 8). In Cluster 2, ICP1_2006_A (HQ641351.1) and ICP1_2006_B (HQ641350.1) have been detected as the top two for the ICP1 (NC_015157.1) therapeutic phage (Supplementary Table 22 and Supplementary Figure 9). ICP2_2011_A (KM224878.1) and JSF27 (KY883658.1) have been found as the top two phages for therapeutic ICP2 (NC_015158.1) phage in Cluster 4 (Supplementary Table 23 and Supplementary Figure 10). As presented in Table 3, JA-1(NC_021540.1) and VCO139 (NC_049350.1) have been found as the top two phages for therapeutic Phi_1 (NC_028799.1) phage in Cluster 5 (Supplementary Figure 11).

**TABLE 3:** Percentage of identity matrix by ClustalOmega for Cluster 5.

	NC_049380.1	NC_028799.1	NC_021540.1	NC_049350.1
NC_049380.1	100	46.47	46.42	46.34
NC_028799.1	46.47	100	**89.79**	**89.65**
NC_021540.1	46.42	**89.79**	100	99.44
NC_049350.1	46.34	**89.65**	99.44	100

*Bold signify the top 2 identity score with respect to the therapeutic phage.*

**FIGURE 6:**
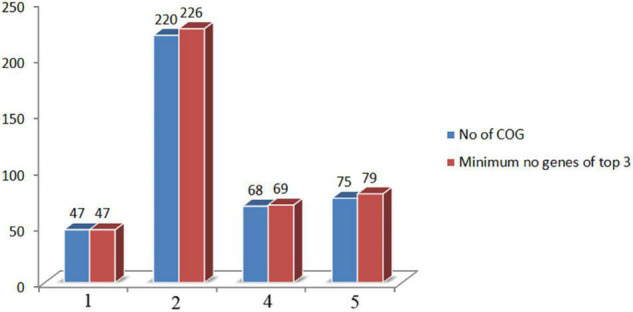
Comparison of orthologous group of proteins and minimum number of encoded proteins among the top three screening phages. COG stands for cluster of orthologous gene.

**FIGURE 7:**
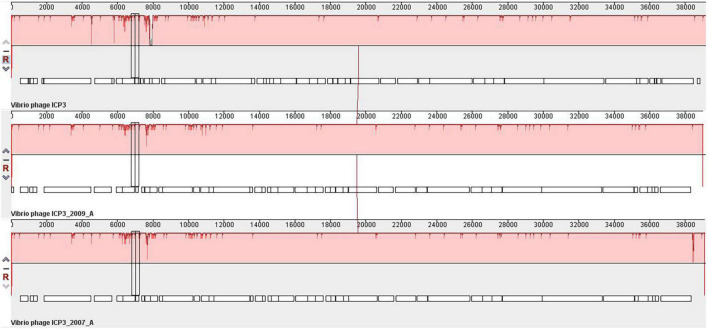
Whole-genome comparative genome analysis of *Vibrio Cholerae* phage ICP3, ICP3_2009_A and ICP3_2007_A using Mauve (Darling et al., 2004).

The orthologous gene investigation of the top three phages of Cluster 1 shows 47 orthologous groups of proteins (Figure 6 and Supplementary Table 24). Forty-two protein sequence level clusters were detected among the ICP3 and ICP3_2007_A using CDHIT with sequence identity cut-off > 90% (Supplementary Table 25). Similarly, 42 clusters were also found among the ICP3 and ICP3_2009_A (Supplementary Table 26). A similar analysis was also carried out for the top three phages of Clusters 2, 4, and 5; 220, 68, and 75 orthologous groups of proteins were found for Clusters 2, 4, and 5, respectively (Figure 6 and Supplementary Tables 27–29). Furthermore, maximum numbers of protein sequence level clusters were also observed for Clusters 2, 4, and 5 using CDHIT with sequence identity cut-off > 90% (Supplementary Tables 27–35). Finally, whole-genome sequences of the top three phages for the above clusters (1, 2, 4, and 5) were also compared and found high similarity among these top three phages for respective clusters (Figure 7 and Supplementary Figures 12–14). However, for ClusterRep, we have not observed any similarity in Mauve (Supplementary Figure 15).

## Discussion

In this study, we have extensively compared 86 *Vibrio cholerae* phages to understand the genomic, functional patterns, and common orthologous proteins among them and their probable usage in cholera therapy. We have noticed that the majority of *Vibrio cholerae* phages are isolated from Bangladesh since the Bengal Delta region is the native homeland of cholera (Figure 1B and Supplementary Table 1; Alam et al., 2011; Kopprio et al., 2020). The genome length and number of predicted proteins of phages displayed a diverse range (Table 1). However, the majority of phage genome length is ∼40 kb and the number of encoded proteins is 40–50. The numbers of encoded proteins are directly proportional to the genome length of phages (Supplementary Table 2) and the majority among them are putative proteins. However, genome length is not a factor to decide the number of tRNAs. For the identification of clusters and singletons, the majority of comparative genomic approaches used whole-genome dot plot and nucleotide identity more than 45–50% at the genus level (Hatfull et al., 2010; Pope et al., 2011; Turner et al., 2017; Ha and Denver, 2018; Gao et al., 2020). Since we have focused on the species level we have considered BLASTn identity and coverage more than 75% within the 86 *Vibrio cholerae* phages for the identification of clusters and singletons (Supplementary Table 2). However, we have verified the clusters using whole-genome dot plot and nucleotide identity and witnessed similar results (Figure 2, Table 2, and Supplementary Tables 3–10). The high- and no-sequence similarities were observed within and between the clusters, respectively (Figure 2 and Supplementary Figure 1). Most of the *Vibrio cholerae* phages were clustered among themselves since we have discovered that 96.5% (83/86 * 100) of phages formed nine clusters (Figure 1A and Supplementary Table 2). Therefore, singletons (3/86, ∼3.5%) are notably lower than *Bacillus* (18.1%), *Pseudomonas* (23.1%), and *Salmonella* (36.6%) (Grose et al., 2014; Ha and Denver, 2018; Gao et al., 2020). We are unable to check the possibility of singleton due to the unique isolation site, since only the isolation country name is retrievable instead of the exact place name. The bias due to the majority (64%) of phages isolated from the same country (Bangladesh) have not reflected the phage diversity and proportion of phages classified into each cluster. As we know that the orthologous proteins function similarly in different species, we have searched for the orthologous group of proteins among the phages. Significant numbers of orthologous groups of proteins have been found within the clusters but no orthologous group of proteins was found between the clusters (ClusterRep). This indicates strong functional similarity and dissimilarity within and between the clusters, respectively (Supplementary Tables 12–20). Furthermore, similarity and dissimilarity within and between the clusters were also clearly visualized by CGView (Figures 3A–D and Supplementary Figures 2–7). We have observed the majority of phages consist of DNA polymerase protein. This protein is liable for DNA replication. The evolutionary analysis of DNA polymerase protein of phages reveals high conservation within the clusters (Figure 4) and high diversity between the clusters (ClusterRep) (shown in Figure 5). In Cluster 2, we have observed that 19 distinct isolates of ICP1 phage are highly conserved, which was earlier found by Angermeyer et al. (2018). For screening of potential therapeutic phages, we have studied only those clusters that should have at least one reported therapeutic phage.

A high degree of sequence similarities within the clusters has been observed in this study. Therefore, screening of potential therapeutic phages from these clusters, we have utilized the guided tree and identity matrix value. The whole-genome sequences of phages have been used to form the guided tree and measure the identity matrix value. The top two similar phages of the therapeutic phage have been identified by guided tree and identity matrix value (Supplementary Tables 21–23 and Supplementary Figures 9–11). A high degree of protein sequence and functional similarity has been observed between the therapeutic and screened phages (Figure 6 and Supplementary Tables 24–29). These indicate strongly that the screening phages might be used for therapeutic targets for *Vibrio cholerae*. The majority of screening phages of Phi_1, ICP1, ICP2, and ICP3 are highly conserved.

We have extensively studied and compared the global database of complete genome sequences of *Vibrio cholerae* phages and testified conserved genomic and functional patterns within the clusters. No conserved genomic and functional patterns have been observed between the clusters. This study can be utilized for the classification of the newly isolated *Vibrio cholerae* phage. A high degree of sequence and functional similarity has been discovered among the therapeutic and screened phages, indicating strong implications in cholera therapy. Furthermore, experimental verification in the animal model might be needed for identifying the exact impact of screened phages in the host *Vibrio cholerae*.

## Data Availability Statement

The original contributions presented in the study are included in the article/Supplementary Material, further inquiries can be directed to the corresponding author/s.

## Author Contributions

AC conceived the study. RB and AC designed and executed the experiments. RB, AC, and SD analyzed the data and wrote the manuscript. All authors contributed to the article and approved the submitted version.

## Conflict of Interest

The authors declare that the research was conducted in the absence of any commercial or financial relationships that could be construed as a potential conflict of interest.

## Publisher’s Note

All claims expressed in this article are solely those of the authors and do not necessarily represent those of their affiliated organizations, or those of the publisher, the editors and the reviewers. Any product that may be evaluated in this article, or claim that may be made by its manufacturer, is not guaranteed or endorsed by the publisher.
