# Supplementary material for: Screening of Potential Vibrio cholerae Bacteriophages for Cholera Therapy: A Comparative Genomic Approach
Source: Front Microbiol. 2022 Mar 29;13:803933. doi: 10.3389/fmicb.2022.803933 (PMC9002330; doi:10.3389/fmicb.2022.803933)
Supplement: Supplementary file 1 [file Data_Sheet_1.ZIP › SupplementaryTablesAndFigures/SupplementaryInformation.docx]

**Supplementary Information**

**Supplementary Tables Legends**

**Table S1.** Primary information of the 86 Vibrio Cholerae phages.

**Table S2.** BLASTn sequence coverage and identity based clusters and their complete informations. Clusters are seperated by single row.

**Table S3.** Average nucleotide identity (ANI) among the first 15 Phages of Cluster 1.

**Table S4.** Average nucleotide identity (ANI) among the first 15 Phages of Cluster 2.

**Table S5.** Average nucleotide identity (ANI) among the Phages of Cluster 3.

**Table S6.** Average nucleotide identity (ANI) among the Phages of Cluster 5.

**Table S7.** Average nucleotide identity (ANI) among the Phages of Cluster 6.

**Table S8.** Average nucleotide identity (ANI) among the Phages of Cluster 7.

**Table S9.** Average nucleotide identity (ANI) among the Phages of Cluster 8.

**Table S10.** Average nucleotide identity (ANI) among the Phages of Cluster 9.

**Table S11.** Average nucleotide identity (ANI) among the Phages of ClusterRep.

**Table S12.** Orthologous groups gene count for Cluster1.

**Table S13.** Orthologous groups gene count for Cluster2.

**Table S14.** Orthologous groups gene count for Cluster3.

**Table S15.** Orthologous groups gene count for Cluster4.

**Table S16.** Orthologous groups gene count for Cluster5.

**Table S17.** Orthologous groups gene count for Cluster6.

**Table S18.** Orthologous groups gene count for Cluster7.

**Table S19.** Orthologous groups gene count for Cluster8.

**Table S20.** Orthologous groups gene count for Cluster9.

**TableS21.** Identity matrix of Cluster 1.

**Table S22.** Identity matrix of Cluster2.

**Table S23.** Identity matrix of Cluster 4.

**Table S24.** Orthologous group of proteins for top 3 phages of Cluster 1.

**Table S25.** Protein sequence level cluster among ICP3 and ICP3_2007_A by CD-HIT.

**Table S26.** Protein sequence level cluster among ICP3 and ICP3_2009_A by CD-HIT.

**Table S27.** Orthologous group of proteins for top 3 phages of Cluster 2.

**Table S28.** Orthologous group of proteins for top 3 phages of Cluster 4.

**Table S29.** Orthologous group of proteins for top 3 phages of Cluster 5.

**Table S30.** Protein sequence level cluster among ICP1 and ICP1_2006_A by CD-HIT.

**Table S31.** Protein sequence level cluster among ICP1 and ICP1_2006_B by CD-HIT.

**Table S32.** Protein sequence level cluster among ICP2 and ICP2_2011_A by CD-HIT.

**Table S33.** Protein sequence level cluster among ICP2 and JSF27 by CD-HIT.

**Table S34.** Protein sequence level cluster among Phi_1 and JA-1 by CD-HIT.

**Table S35.** Protein sequence level cluster among Phi_1 and VCO139 by CD-HIT.

**Supplementary Figures Legends**

**Figure S1.** Dot plot of 12 Vibrio Cholerae phages present in ClusterRep. Dot plot is visualized by Gepard version 1.30.

**Figure S2.** Comparative whole-genome map of ICP 1 phage and Cluster 2 phages.

**Figure S3.** Comparative whole-genome map of J2 phage and Cluster 3 phages.

**Figure S4.** Comparative whole-genome map of JA-1 phage and Cluster 5 phages.

**Figure S5.** Comparative whole-genome map of JSF10 phage and Cluster 7 phages.

**Figure S6.** Comparative whole-genome map of CP-T1 phage and Cluster 8 phages.

**Figure S7.** Comparative whole-genome map of X29 phage and Cluster 9 phages.

**Figure S8.** Screening of potential therapeutic phages for Cluster 1.

**Figure S9.** Screening of potential therapeutic phages for Cluster 2.

**Figure S10.** Screening of potential therapeutic phages for Cluster 4.

**Figure S11.** Screening of potential therapeutic phages for Cluster 5.

**Figure S12.** Whole-genome comparative analysis of *Vibrio Cholerae* phage ICP1, ICP1_2006_A and ICP1_2006_B using Mauve.

**Figure S13.** Whole-genome comparative analysis of *Vibrio Cholerae* phage ICP2, ICP2_2011_A and JSF27 using Mauve.

**Figure S14.** Whole-genome comparative analysis of *Vibrio Cholerae* phage Phi 1, JA_1 and VCO139 using Mauve.

**Figure S15.** Whole-genome comparative analysis of *Vibrio Cholerae* phage ICP2, ICP3 and Phi-1 using Mauve.
