# Supplementary figures and images for: Screening of Potential Vibrio cholerae Bacteriophages for Cholera Therapy: A Comparative Genomic Approach
Source: Front Microbiol. 2022 Mar 29;13:803933. doi: 10.3389/fmicb.2022.803933 (PMC9002330; doi:10.3389/fmicb.2022.803933)

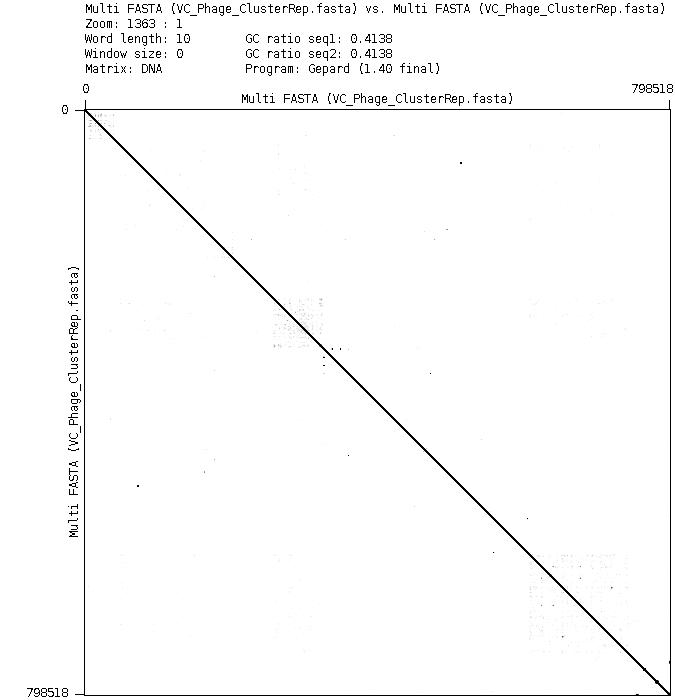

Supplement: Supplementary file 1 [file Data_Sheet_1.ZIP › SupplementaryTablesAndFigures/FigureS1.tif]

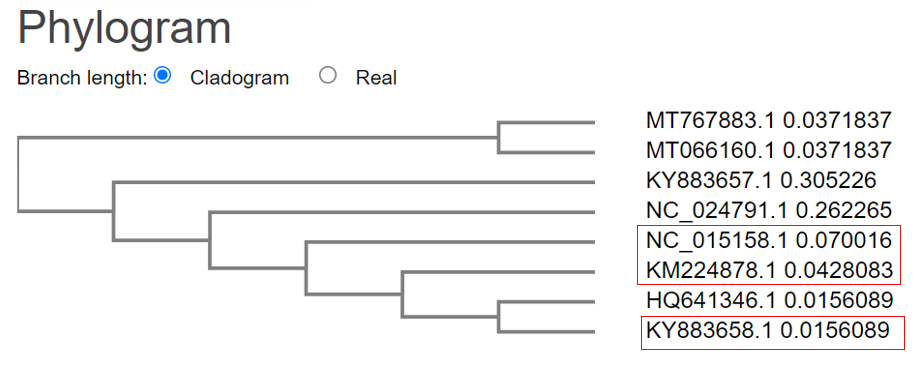

Supplement: Supplementary file 1 [file Data_Sheet_1.ZIP › SupplementaryTablesAndFigures/FigureS10.tif]

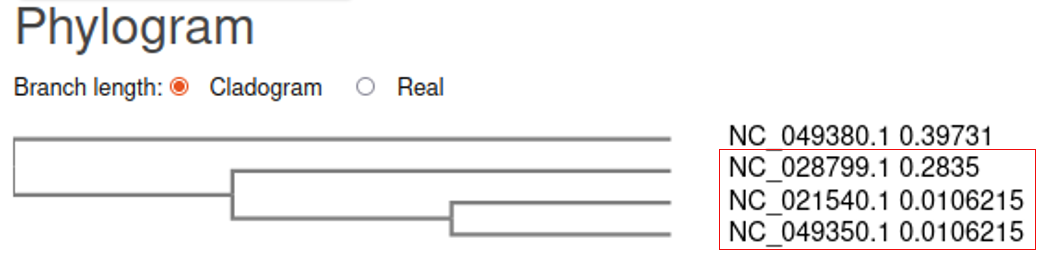

Supplement: Supplementary file 1 [file Data_Sheet_1.ZIP › SupplementaryTablesAndFigures/FigureS11.tif]

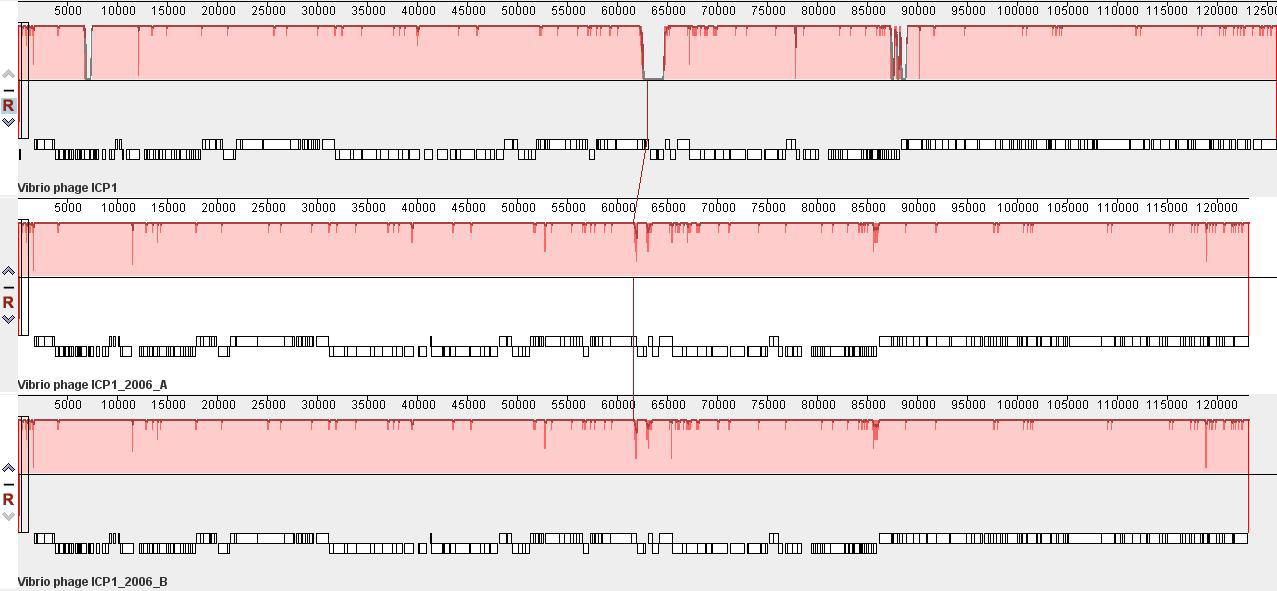

Supplement: Supplementary file 1 [file Data_Sheet_1.ZIP › SupplementaryTablesAndFigures/FigureS12.tif]

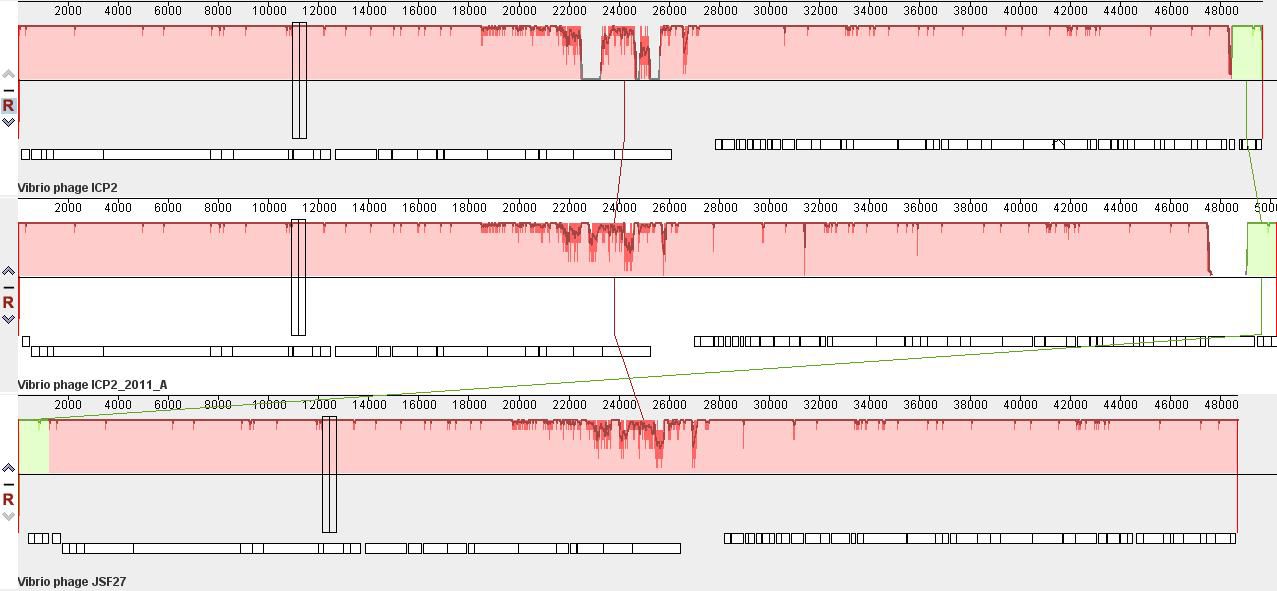

Supplement: Supplementary file 1 [file Data_Sheet_1.ZIP › SupplementaryTablesAndFigures/FigureS13.tif]

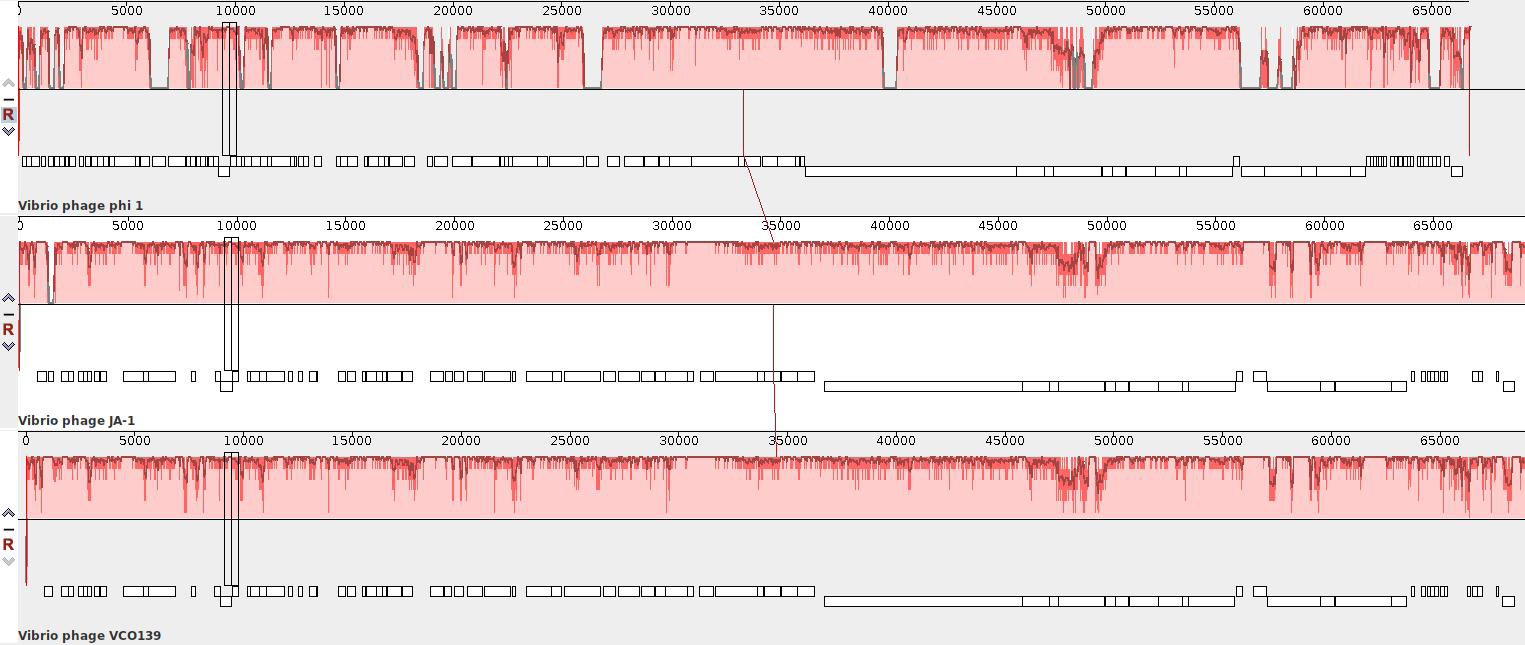

Supplement: Supplementary file 1 [file Data_Sheet_1.ZIP › SupplementaryTablesAndFigures/FigureS14.tif]

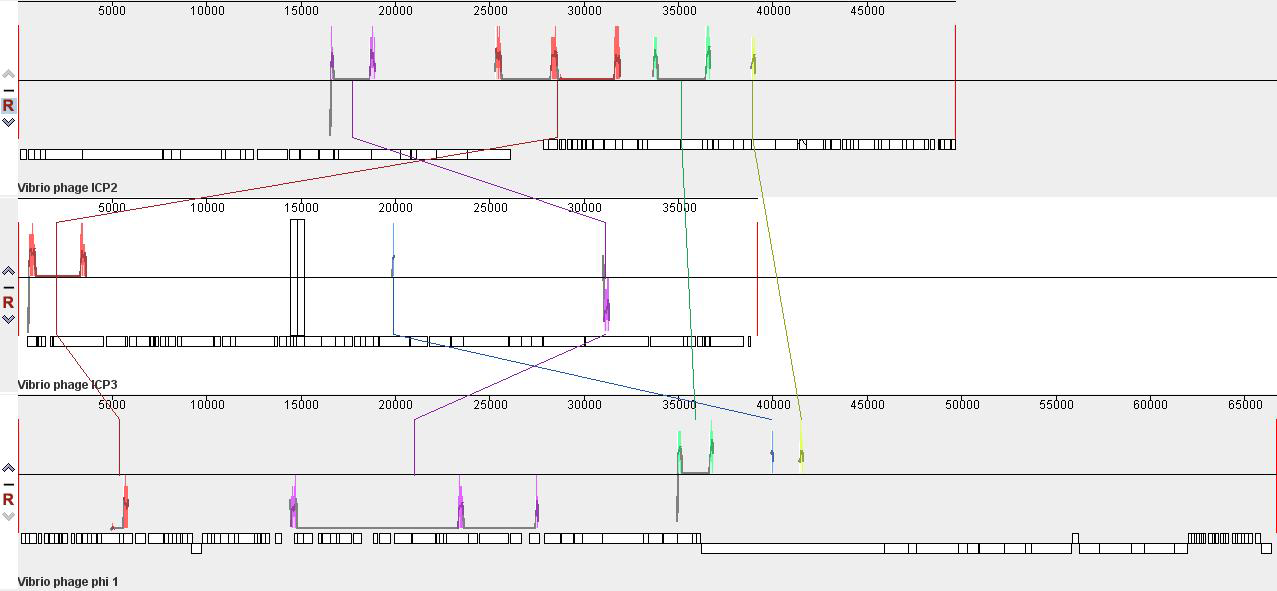

Supplement: Supplementary file 1 [file Data_Sheet_1.ZIP › SupplementaryTablesAndFigures/FigureS15.tif]

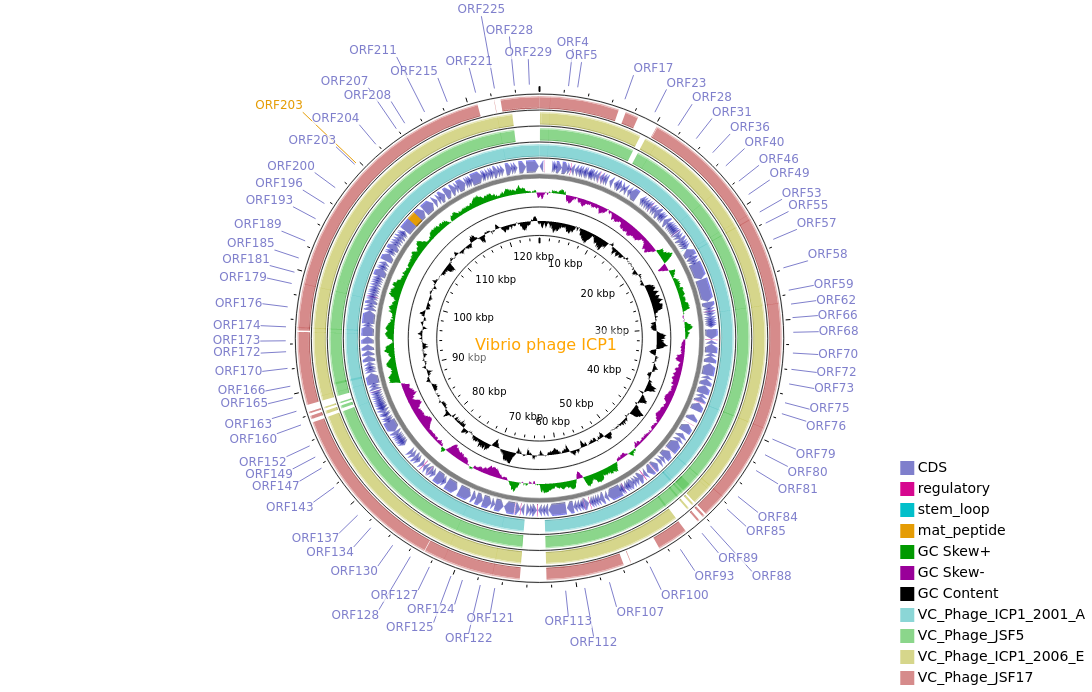

Supplement: Supplementary file 1 [file Data_Sheet_1.ZIP › SupplementaryTablesAndFigures/FigureS2.tif]

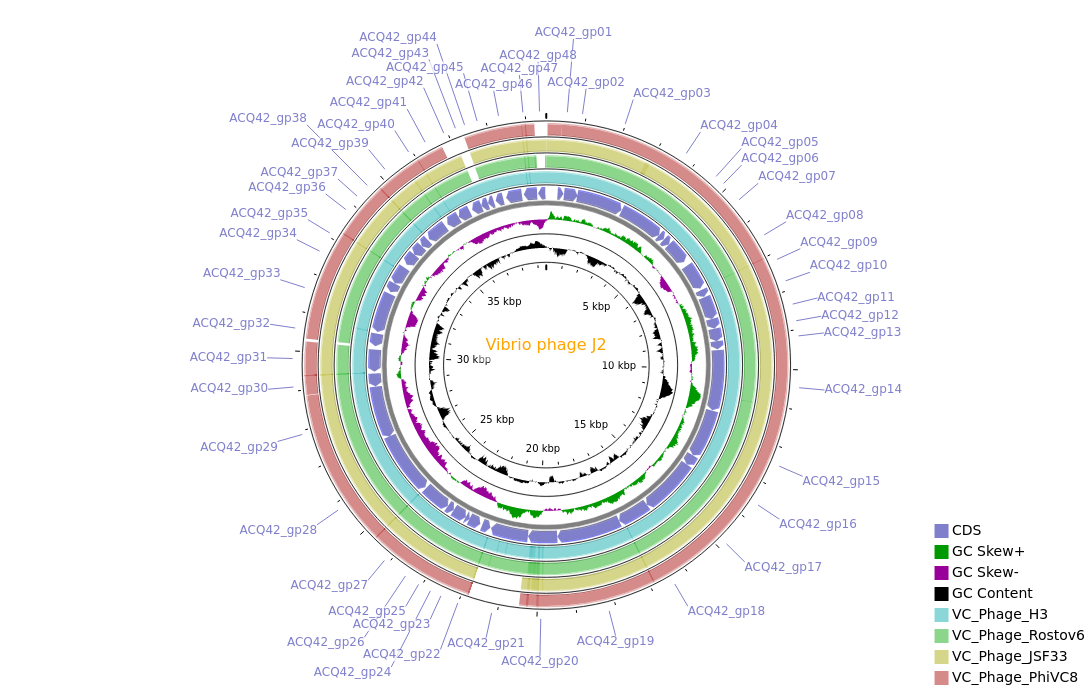

Supplement: Supplementary file 1 [file Data_Sheet_1.ZIP › SupplementaryTablesAndFigures/FigureS3.tif]

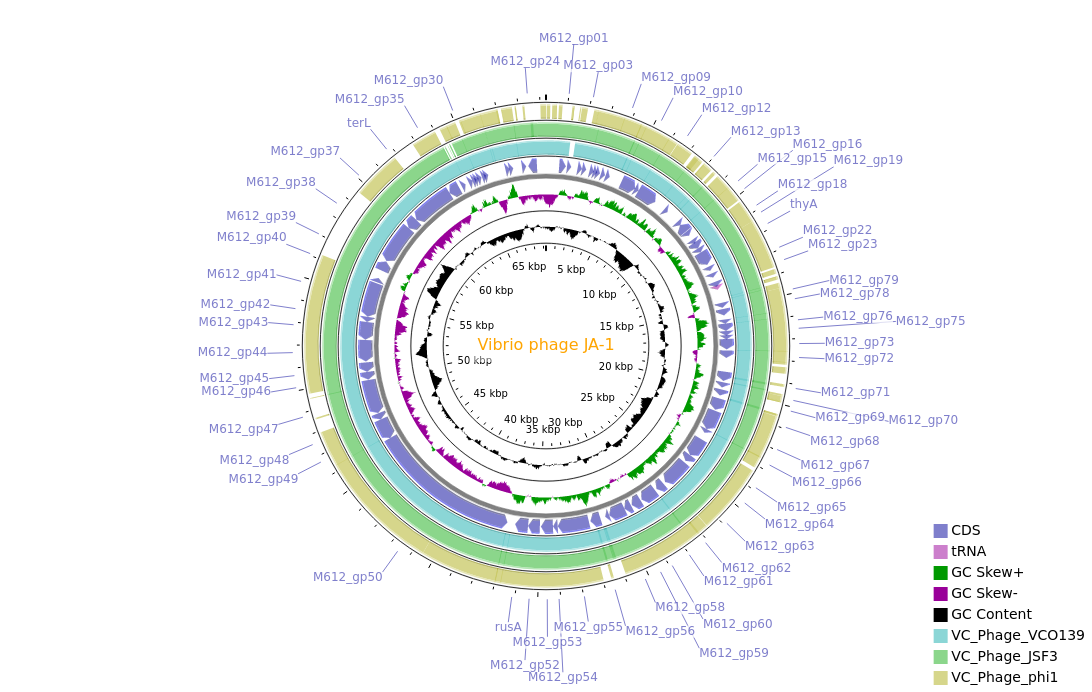

Supplement: Supplementary file 1 [file Data_Sheet_1.ZIP › SupplementaryTablesAndFigures/FigureS4.tif]

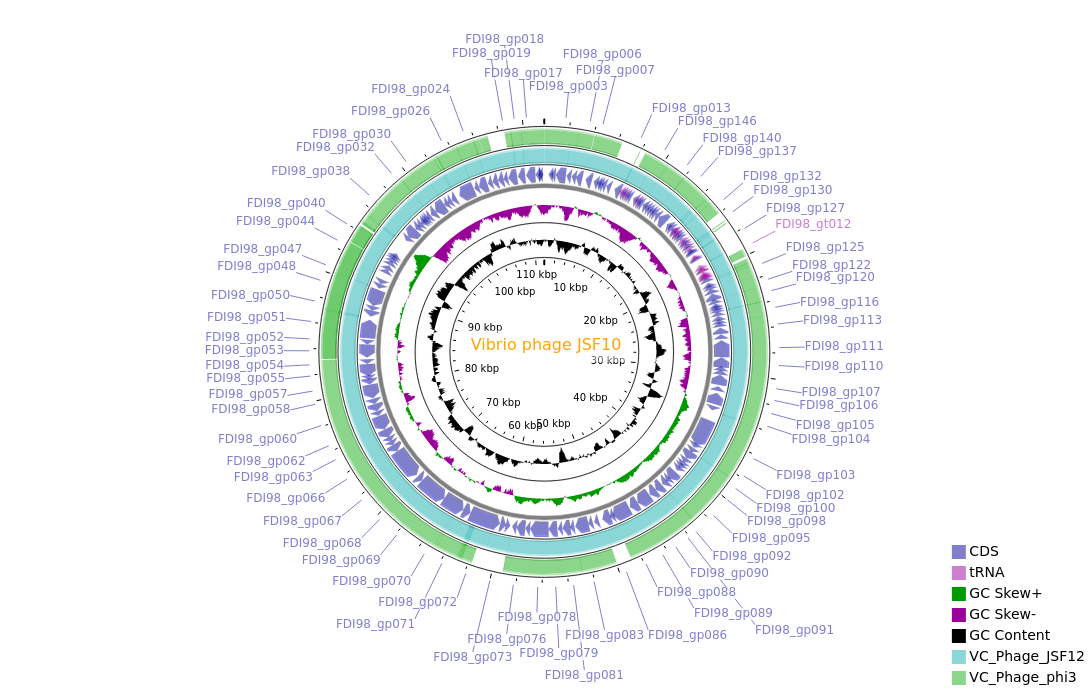

Supplement: Supplementary file 1 [file Data_Sheet_1.ZIP › SupplementaryTablesAndFigures/FigureS5.tif]

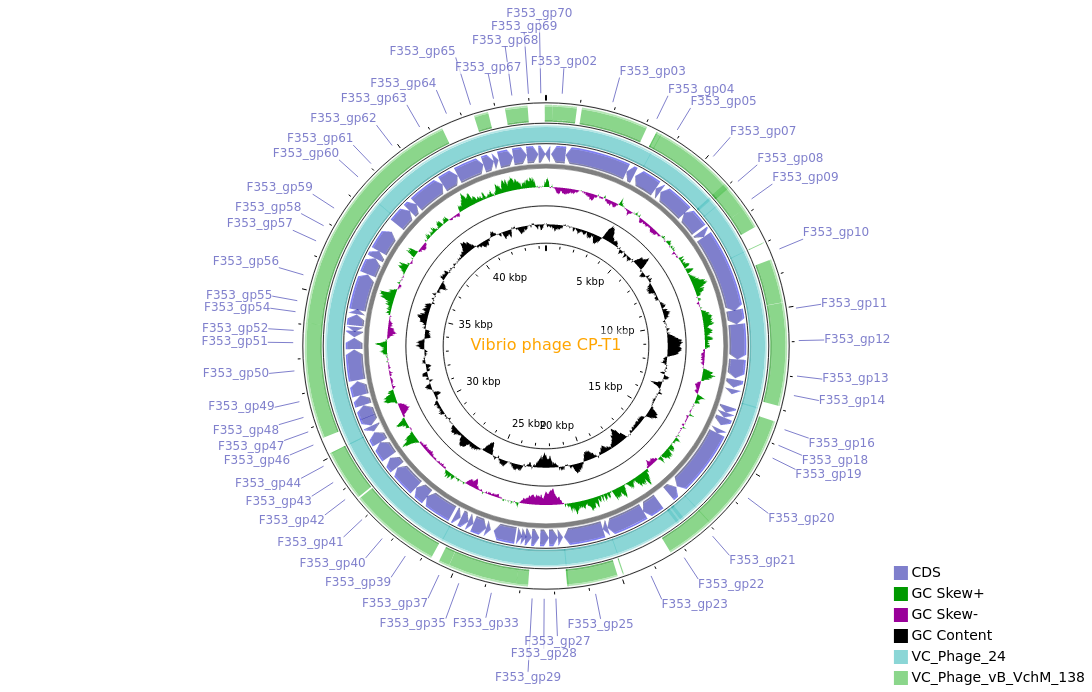

Supplement: Supplementary file 1 [file Data_Sheet_1.ZIP › SupplementaryTablesAndFigures/FigureS6.tif]

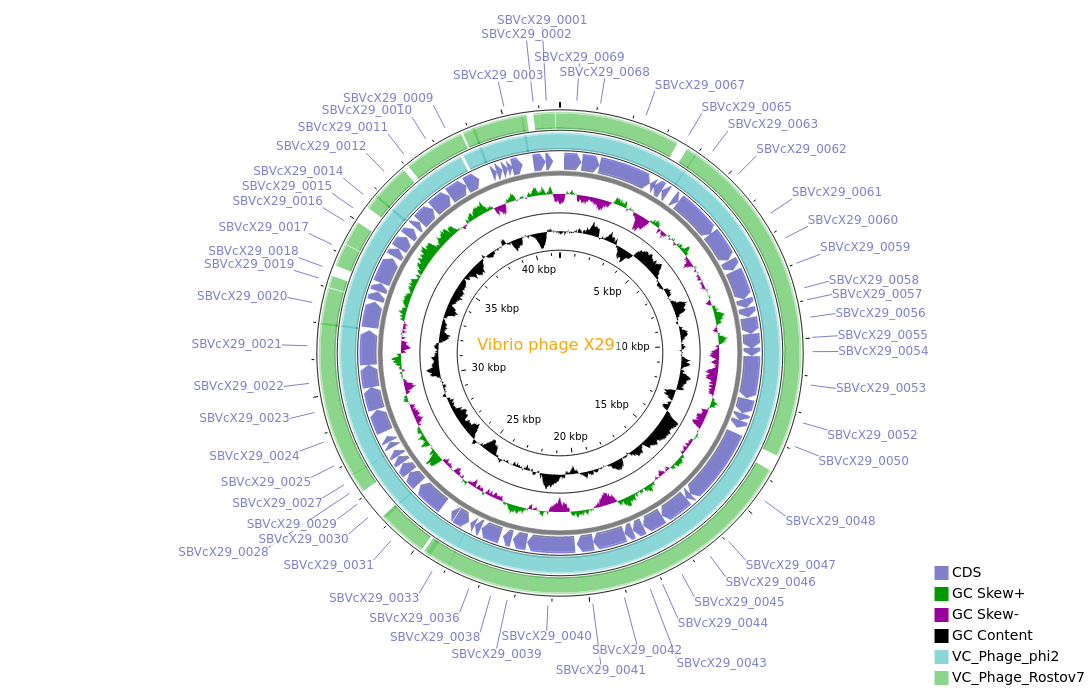

Supplement: Supplementary file 1 [file Data_Sheet_1.ZIP › SupplementaryTablesAndFigures/FigureS7.tif]

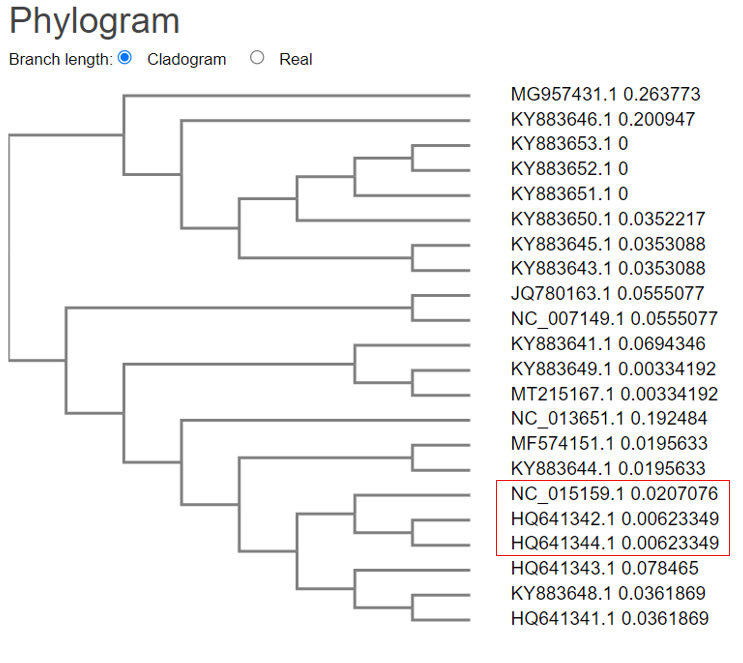

Supplement: Supplementary file 1 [file Data_Sheet_1.ZIP › SupplementaryTablesAndFigures/FigureS8.tif]

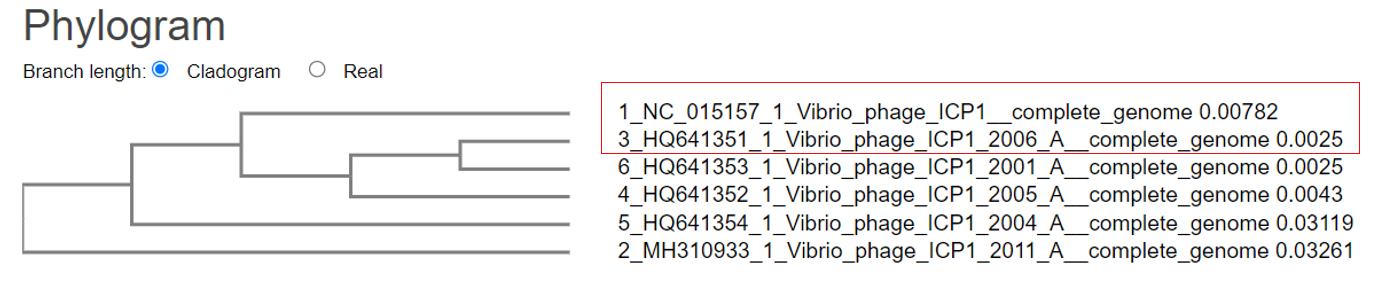

Supplement: Supplementary file 1 [file Data_Sheet_1.ZIP › SupplementaryTablesAndFigures/FigureS9A.tif]

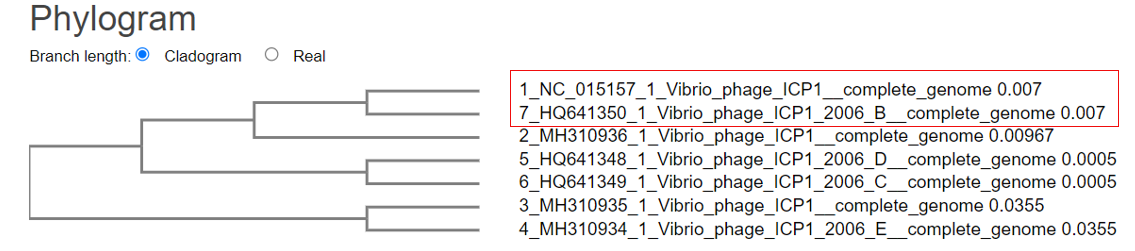

Supplement: Supplementary file 1 [file Data_Sheet_1.ZIP › SupplementaryTablesAndFigures/FigureS9B.tif]
